# Erratum for Betancourt et al., “Pulmonary granuloma formation during latent *Cryptococcus neoformans* infection in C3HeB/FeJ mice involves progression through three immunological phases”

**DOI:** 10.1128/mbio.00595-25

**Published:** 2025-03-12

**Authors:** Jovany J. Betancourt, Minna Ding, J. Marina Yoder, Issa Mutyaba, Hannah M. Atkins, Gabriela De la Cruz, David B. Meya, Kirsten Nielsen

## ERRATUM

Volume 16, no. 2, e03610-24, 2025, https://doi.org/10.1128/mbio.03610-24. Page 16, Acknowledgments, second paragraph, first sentence: “R01AI34636” should read “R01AI176922.”

