# Supplementary material for: Pulmonary granuloma formation during latent Cryptococcus neoformans infection in C3HeB/FeJ mice involves progression through three immunological phases
Source: mBio. 2025 Jan 14;16(2):e03610-24. doi: 10.1128/mbio.03610-24 (PMC11796415; doi:10.1128/mbio.03610-24)
Supplement: Supplemental Figures — Figures S1 to S4. [file mbio.03610-24-s0001.pdf]

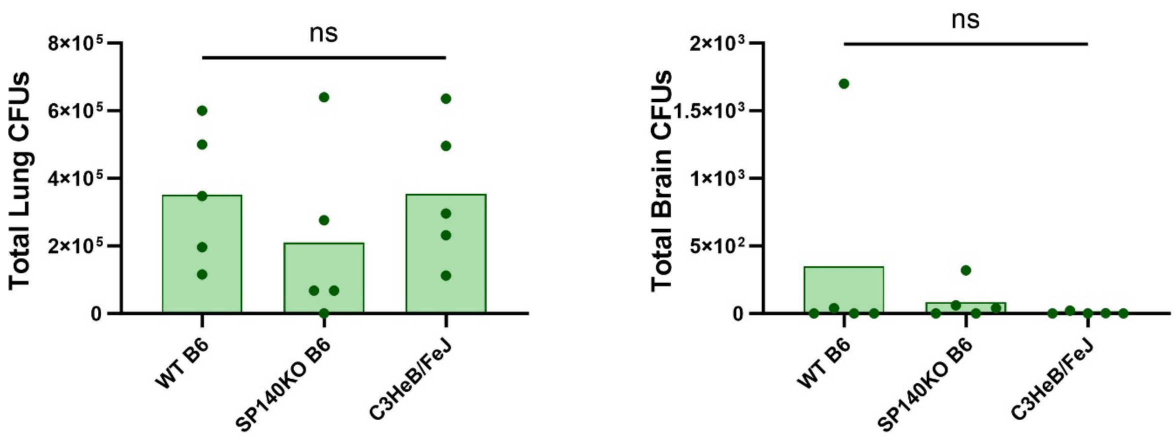

Supplemental Figure 1: SP140 deficiency does not influence lung or brain fungal burden. Lungs and brains from UgCl223-infected WT-B6, SP140$^{-/-}$ B6 (SP140KO B6), and C3HeB/FeJ mice were excised after 30 DPI, plated on YPD agar, and *C. neoformans* colonies enumerated after 48 hours of incubation. Ordinary one-way ANOVA with Tukey's post-hoc analysis was performed. n = 5. ns = non-significant.

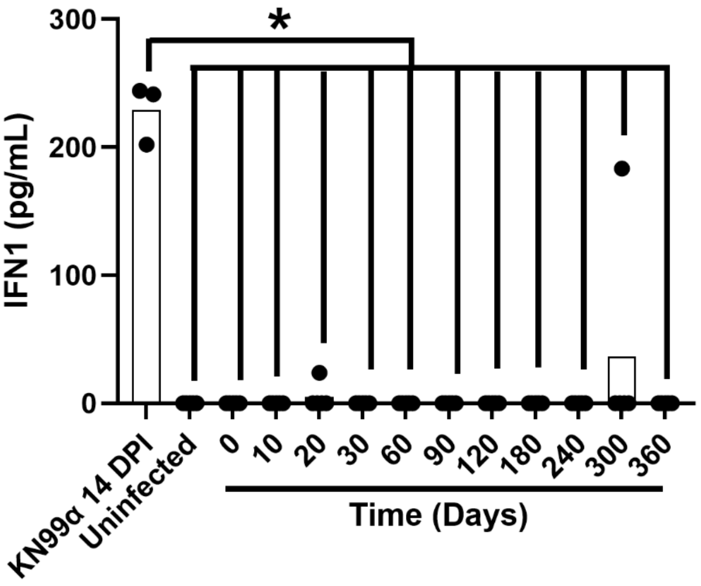

Supplemental Figure 2: IFN1 abundance is lower in latent cryptococcus infection relative to lethal KN99α infection. Lung serum from C3HeB/FeJ mice infected with either KN99α at 14 DPI or UgCl223 over 360 DPI were analyzed for IFN1 abundance using a multiplex immunoassay. Ordinary one-way ANOVA with Tukey's post-hoc. * = p-value <0.05.

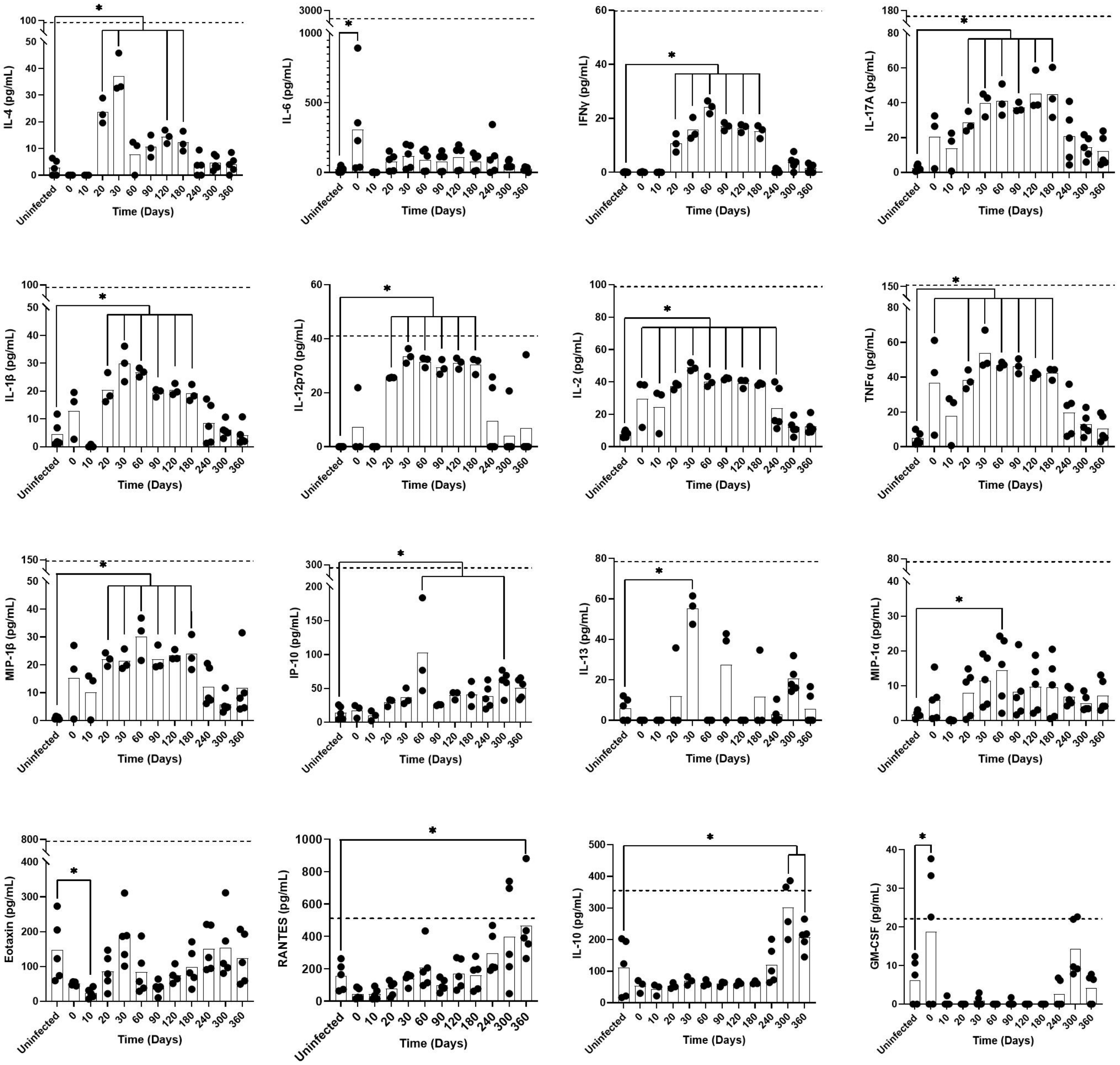

Supplemental Figure 3: Individual cytokine concentrations during latent infection. Lung serum from homogenized tissue was isolated from C3HeB/FeJ mice latently infected with UgCl223 and analyzed by multiplex assay for cytokine abundance. Lung serum was analyzed at designated time points from 0-360 days. The dashed lines represent the mean cytokine concentration for the KN99α infection at 20 days post infection. Ordinary one-way ANOVA comparing means to uninfected. * = p-value ≤ 0.05.

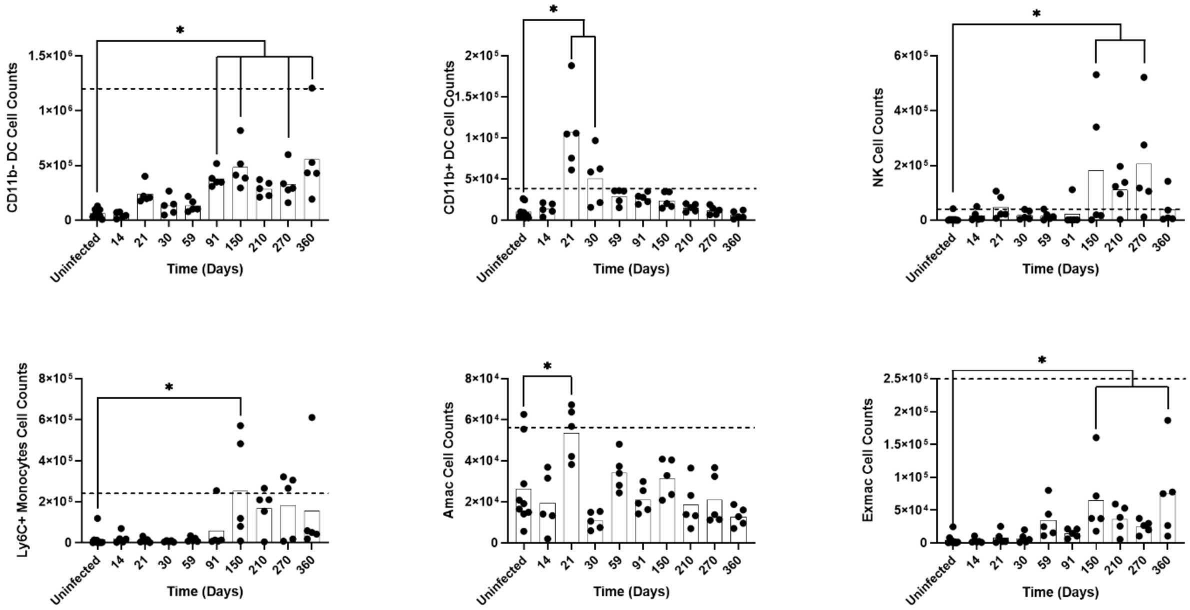

Supplemental Figure 4: Cell counts for bulk immune cell flow cytometry panel. Pulmonary immune cells were isolated from C3HeB/FeJ mice latently infected with UgCl223 and analyzed by flow cytometry. Cells were analyzed at designated time points from 0-360 days and enumerated. The dashed lines represent the mean cell counts for the KN99α infection at 14 days post infection. Ordinary one-way ANOVA comparing means to uninfected. * = p-value ≤ 0.05.
